# Prostatic Artery Embolization (PAE) Using Polyethylene Glycol Microspheres: Safety and Efficacy in 81 Patients

**DOI:** 10.1007/s00270-022-03165-4

**Published:** 2022-06-02

**Authors:** Iñigo Insausti, Arkaitz Galbete, Vanesa Lucas-Cava, Ana Sáez de Ocáriz, Saioa Solchaga, Raquel Monreal, Antonio Martínez de la Cuesta, Raquel Alfaro, Fei Sun, Manuel Montesino, Fermin Urtasun, José Ignacio Bilbao Jaureguízar

**Affiliations:** 1 Department of Interventional Radiology, Hospital Universitario de Navarra, Pamplona, Spain; 2 Universidad Pública de Navarra–Navarrabiomed–Hospital Universitario de Navarra, Redissec, Instituto de Investigacion Sanitaria de Navarra (IdiSNA), Pamplona, Spain; 3 Endoluminal Therapy and Diagnosis Unit, Jesús Usón Minimally Invasive Surgery Centre, Cáceres, Spain; 4 Department of Interventional Radiology, Clinica Universidad de Navarra, Pamplona, Spain; 5 Servicio Navarro de Salud, Osasunbidea, Pamplona, Spain; 6 Department of Urology. Hospital, Universitario de Navarra, Pamplona, Spain

**Keywords:** Prostatic hyperplasia, Lower urinary tract symptoms, Prostatic artery embolization

## Abstract

**Purpose:**

To evaluate the safety and efficacy of prostatic artery embolization (PAE) using polyethylene glycol microspheres (PEGM) in patients with lower urinary tract symptoms secondary to benign prostatic hyperplasia (BPH).

**Materials and methods:**

This multicentric prospective study enrolled 81 patients who underwent PAE with 400 ± 75 µm PEGM (HydroPearl®, Terumo, Japan). Results from baseline and 1-, 3-, 6-, and 12-month follow-ups were assessed for subjective outcomes including International Prostate Symptoms Score (IPSS), Quality of life (QoL), and International Index of Erectile Function, and objective outcomes such as peak urinary flow (Qmax) and post-void residual volume (PVR). The visual analogue scale, satisfaction questionnaire, prostatic volume, and prostatic specific antigen levels were also evaluated. Complications were documented using the modified Clavien–Dindo classification.

**Results:**

Technical success was obtained in all patients. Clinical success was achieved in 78.5% of patients. Before PAE, 54.3% of patients had an indwelling catheter which was removed in 75% of them after procedure. A statistically significant decrease was observed in IPSS and QoL from baseline to 12 months (20.14 vs 5.89; 4.8 vs 0.63, *P* < .01), respectively. Objective outcomes also showed a statistically significant improvement in Qmax (+ 114.9%; *P* < .01), achieving a maximum urinary flow of 14.2 mL/sec, and PVR (decrease 58%; *P* < .05) at 12 months. Minor complications (Clavien–Dindo grades I–II) occurred in 13.6% of patients, without major complications observed.

**Conclusion:**

PAE with PEGM is safe and effective treatment in patients with symptomatic BPH, with a significant improvement in both subjective and objective outcomes.

## Introduction

Prostatic artery embolization (PAE) as a minimally invasive technique has been widely used in the management of lower urinary tract symptoms (LUTS) secondary to benign prostatic hyperplasia (BPH) during the last decade. Recent systematic reviews and meta-analyses have demonstrated that PAE is efficacious and safe to improve functional parameters such as the International Prostate Symptom Score (IPSS), quality of life (QOL), peak urinary flow rate (Qmax), and post-void residual volume (PVR) after PAE and can significantly relieve LUTS for 24 months [1–3].

As new embolic agents continue to be developed, the use of various embolic particles with different particle sizes in PAE has been reported in clinical practice, including non-spherical polyvinyl alcohol (PVA) particles (Contour), spherical PVA (Bead Block), trisacryl gelatin microspheres (Embosphere), and polyzene-coated hydrogel microspheres (Embozene) [4–10]. Recently, an embolic agent, polyethylene glycol microspheres (PEGM, HydroPearl) was cleared for use in PAE procedure by the US Food and Drug Administration in January 2020.

HydroPearl microspheres are spherical, biocompatible, and non-resorbable particles, which are engineered for accurate embolization at the target area. Compared to the commonly used Embosphere in PAE, HydroPearl microspheres are less rigid and more compressible with a higher deformation in the vitro study. These characteristics of PEGM are believed to have the potential to induce more distal occlusion within the prostatic vasculature, thus enhancing the therapeutic effect in PAE [11]. Another feature of HydroPearl microspheres is the tight calibration with more than 90% of the particles falling within the size range as indicated on the labels (e.g., 400 ± 75 µm), resulting in tighter luminal packing when delivered into target vessels, and ultimately causing greater tissue necrosis [12]. Although PAE with PEGM has shown promising results in the canine model [13], the safety and efficacy of PEGM used in PAE remain to be proven clinical practice. The aim of this study was to assess the effectiveness and safety of PAE with PEGM for LUTS secondary to BPH.

## Materials and Methods

### Study Design and Patients

This multi-center prospective cohort study was conducted in accordance with the Declaration of Helsinki [14] and the standards of Good Clinical Practice [15], with approval from the institutional review board. Informed consent was obtained from all patients. The study was conducted by the Interventional Radiology and Urology Departments of the Hospital Universitario de Navarra. This article follows the Strengthening the Reporting of Observational Studies in Epidemiology (STROBE) recommendations for reporting observational studies [16].

Previously, all patients were evaluated by the urologist and were obtained a complete medical history, physical examination including rectal examination, IPSS, QoL, and sexual function using the International Index of Erectile Function (IIEF) questionnaire. Uroflowmetry to measure Qmax, and blood tests for prostate-specific antigen (PSA) were also performed. Imaging studies prior to the procedure included transabdominal ultrasound (US), computed tomography (CT) angiography, and multiparametric magnetic resonance imaging (mpMRI). PVR was measured by transabdominal US. Prostate volume (PV) was assessed by mpMRI. Prostate biopsy was performed whenever a suspicious focal lesion was detected on mpMRI or digital rectal examination, or when the PSA was > 4 mg/mL.

From July 2015 to March 2020, 92 patients with LUTS secondary to BPH were assessed for study eligibility. The inclusion criteria were age > 40 years; PV ≥ 30 cm^3^; BPH-related LUTS refractory to medical treatment for at least 6 months or intolerance to medical treatment; IPSS > 8 points; QoL ≥ 3 points; Qmax ≤ 12 mL/sec, or urinary retention. The exclusion criteria included the presence of advanced atherosclerosis and tortuosity of the iliac arteries, non-visualization of the prostatic artery or other accessory arteries supplying the prostate on CT angiography, glomerular filtration rate of less than 30 mL/min, acute infection (e.g., prostatitis), the presence of prostate cancer, and previous diagnostic of urethral stenosis, detrusor muscle failure or neurogenic bladder.

From 92 patients evaluated for eligibility, 11 patients were excluded due to the presence of prostate cancer (*n* = 2), advanced atherosclerosis (*n* = 7), and neurogenic bladder (*n* = 2). Finally, 81 patients were enrolled, of whom 79 patients were followed up at least once. Clinical follow-up could not be performed in two patients, one lost to follow-up and a further one deceased by ischemic stroke unrelated to the procedure. Patients were evaluated in the urology consultation at 3, 6, and 12 months to assess BPH medication discontinuation.

### Procedure

PAE was performed by 3 interventional radiologists (I.I., A.S.O., S.S.; with 8, 7 and 6 years of experience in this technique, respectively). Embolization was performed under local anesthesia via unilateral femoral approach, most commonly right, and only in a few patients via left radial access. A 5-F hydrophilic Roberts uterine catheter (Cook Medical, Bloomington, Indiana) or Cobra 1 catheter (Terumo, Tokyo, Japan) were used to catheterize both internal iliac arteries. All arteries supplying the prostate were catheterized using a Progreat α 2.0 microcatheter (Terumo), a 0.014-inch Synchro Soft guidewire (Stryker Neuro-vascular, Fremont, California), 0.014–0.016-inch Glidewire GT double-angled or a 90º-angled micro–guide wire (Terumo). When the microcatheter was selectively placed into the prostatic artery, embolization was carried out using 400 ± 75 µm PEGM (HydroPearl®, Terumo, Japan). The microspheres were delivered in 20-mL syringes containing 2 mL of microspheres diluted with 4 mL of NaCl and mixed with 3 mL of contrast (Visipaque 320, GE Healthcare, Little Chalfont, UK) and 1 mL of saline to achieve an adequate particle suspension. The embolization end point was achieved when stasis of contrast with disruption of arterial flow and opacification of prostate gland were present.

Most patients were discharged within 24 h following PAE, and some patients were discharged 4–5 h after the procedure when radial access or femoral closure device was used.

### Outcomes

Primary outcomes assessed were QoL, IPSS, and Qmax, and the secondary outcomes were PV, IIEF, PVR, and PSA. All outcomes were measured before the procedure, and at 1-, 3-, 6-, and 12-month follow-ups, except in Qmax and PVR which were measured before the procedure, and at 3, 6, and 12 months.

In addition, the presence of prostate infarction as well as the volume of infarction was assessed by mpMRI at one month after PAE. The volume of ischemia tissue was calculated on contrast-enhanced T1-weighted images using Philips IntelliSpace Portal 3D volume software (Amsterdam, the Netherlands).

Technical success was defined as embolization of at least one prostatic artery. Clinical success included the presence of the following [17]: a decrease of at least 25% in IPSS from baseline with a final IPSS score ≤ 18 points; a decrease of at least 1 point in QoL score from baseline with a final score of ≤ 3 points; removal of the indwelling catheter with subsequent spontaneous micturition in patients with a urinary catheter before PAE.

Safety of PAE was assessed by the number and severity of adverse events, which were graded according to modified Clavien–Dindo classification system [18]. Pain and satisfaction with the procedure were measured at 24 h following PAE by phone call, as described in previous studies [19]. Other complementary parameters were assessed such as arterial access site, type of anesthesia, fluoroscopy time, radiation dose, length of post-procedure hospital stay, and interruption of medical therapy.

### Statistical Analysis

Descriptive analysis was performed and expressed by mean and standard deviation for quantitative variables and by frequency and percentage for categorical variables. The statistical differences at different follow-up time-points were analyzed using student *t*-test for paired data, showing mean difference from baseline with 95% confidence interval and percentage change. All comparisons were two-sided with a significance level of 0.05.

## Results

### Baseline Characteristics

The baseline characteristics of the included patients are shown in Table 1. The mean age was 73.87 ± 10.21 years (range, 54–92 years) with IPSS 20.14 ± 4.33 points, PV 96.24 ± 47.1 ml, and Qmax 6.99 ± 2.8 mL/s. The most frequent symptom in urology consultation was urine retention. Prior to PAE, 92.6% (75/81) of the patients were on medical treatment for LUTS secondary to BPH.

**Table 1:** Baseline patient data

Variable	PAE (*n* = 81)
Age (years)	73.87 (10.2)
LUTS (number of patients, %)	
Urinary retention	(44) 54.3%
Weak or interrupted stream	(28) 34.6%
Intermittency	(22) 27.2%
Nocturia	(14) 17.3%
Incomplete emptying	(12) 14.8%
Others	(12) 14.8%
Previous medical therapy (number of patients, %)	(75) 92.6%
α-1 ARA + 5-ARI	(57) 70.4%
α-1 ARA monotherapy	(15) 18.5%
α-1 ARA + anticholinergics	(3) 3.7%
No medical therapy	(6) 7.4%
PV (cm^3^)	96.24 (47.1)
PSA (ng/dL)	7.21 (11.6)
Urinary catheter prior to PAE (number of patients, %)	44 (54.3%)
IPSS (points)	20.14 (4.33)
QoL (points)	4.8 (1.1)
Qmax (mL/s)	6.99 (2.8)
PVR (mL)	147.04 (184.3)
IIEF (points)	5.84 (9.2)

Data are mean (standard deviation) unless indicated otherwise

*LUTS* lower urinary tract symptoms, *α*1*-ARA* α1-adrenergic receptor antagonist, 5*-ARI* 5-α reductase inhibitor, *PV* prostate volume, *PSA* prostate-specific antigen, *IPSS* international prostate symptom score, *QoL* quality of life, *Qmax* peak urinary flow rate, *PVR* post-void residual, *IIEF* international Index of erectile dysfunction

### Perioperative and Postoperative Data

Perioperative and postoperative data are shown in Table 2. Technical success was obtained in 100% (81/81) of the patients. Bilateral PAE was performed in 69 (85.2%) patients, whereas unilateral PAE was due to the impossibility of catheterization of the prostate artery (*n* = 3), the presence of anastomoses to the rectum or penis not suitable for embolization (*n* = 4), and sub-occlusive arterial atheromatous disease (*n* = 5).

**Table 2:** Perioperative and postoperative data

Variable	PAE (*n* = 81)
Access (number of patients, %)	
Femoral	78 (96.3%)
Radial	3 (3.7%)
Anesthesia (number of patients, %)	
General	1 (1.2%)
Regional	0 (0%)
Local	80 (98.8%)
Embolization (number of patients, %)	
Bilateral	69 (85.2%)
Unilateral	12 (14.8%)
Injected microspheres (mL)^a^	2.65 (1.71)
Procedure time (min)	135.13 (76.24)
Fluoroscopy time(min)	41.82 (26.57)
DAP (Gy/cm^2^)	203.2 (38.6)
Pain at 24 h (VAS) ^e^	1.24 (0.44)
Satisfaction at 24 h (points)^be^	81.4 (10.2)
Length of hospital stay (days)	0.85 (0.36)
Prostatic infarct^c^	64.4%
Infarct volume (cm^3^)^d^	9.4 (1.5)

Data are mean (standard deviation) unless indicated otherwise

*PAE* prostate artery embolization, *VAS* visual analogue scale, *DAP* dose area product

^a^injected microspheres (mL) in each prostatic artery

^b^satisfaction with the procedure was measured on a scale of 0 (very dissatisfied) to 100 (very satisfied) at 24 h after PAE

^c^Percentage of patients with prostatic infarct seen in 1^st^ month MRI

^d^Infarct volume calculated on post-embolisation T1-weighted post-contrast-enhanced MRI images at 1^st^ month

^e^Procedure-related pain and satisfaction were assessed by a telephone call to the patient 24 h after PAE

The mean procedure time was 135.13 ± 76.24 min, and the mean fluoroscopy time was 41.82 ± 26.57 min. The mean dose radiation was 203.2 ± 38.6 Gy/cm^2^. The mean amount of microspheres injected into each prostate artery was 2.65 ml (range, 1–6 ml).

Regarding hospital stay, 69 patients (85.2%) were admitted for 24 h after PAE, and 12 patients (14.8%) were treated on an outpatient basis. All patients were discharged without immediate complications. The mean PSA at 24 h was 77.68 ng/dL (range 0.01–2000 ng/dL). Furthermore, pain measured by visual analogue scale (VAS) was 1.24 ± 0.44 points, and the mean satisfaction measured on a scale of 0 (very dissatisfied) to 100 (very satisfied) was of 81.4 ± 10.2 points at 24 h after PAE. The percentage of patients who stopped taking medication for LUTS secondary to BPH after PAE was 91.3% at 3 months, 95% at 6 months, and 97.5% at 12 months. Only 2.5% of the patients continued with medical treatment at 12 months.

### Primary and Secondary Outcomes

**Fig. 1:**
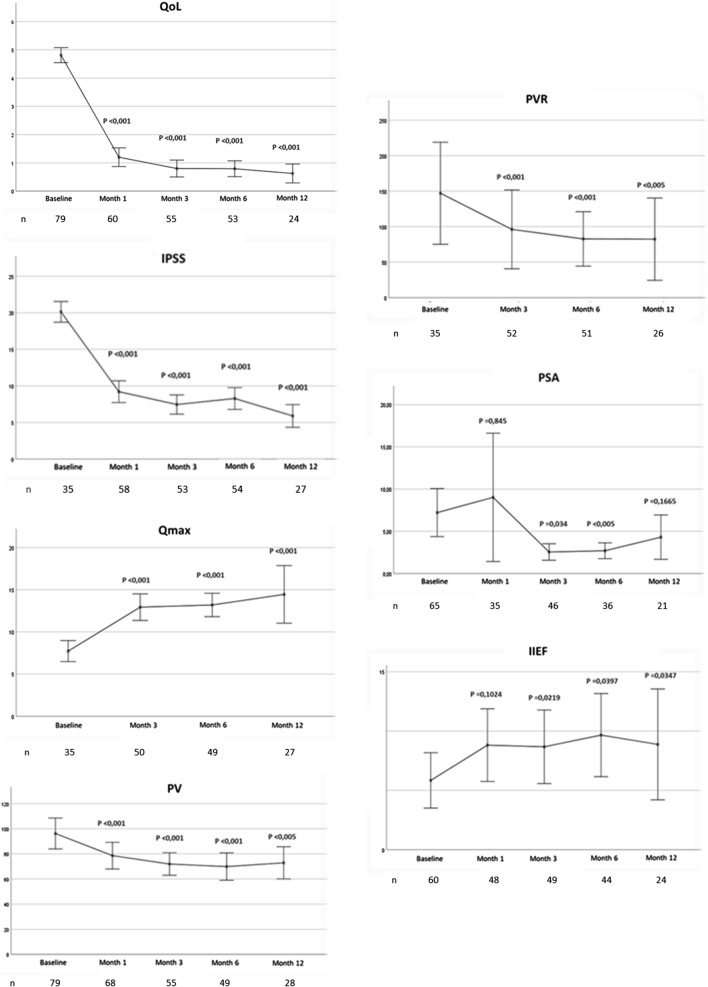
Longitudinal outcome data during the follow-up

Primary and secondary outcome data are shown in Table 3 and Fig. 1. Statistically significant improvements at 12 months of follow-up compared with baseline data were observed in IPSS (reduction of 63.3%, *P* < 0.01), in QoL (reduction of 85%, *P* < 0.01), in Qmax (increase of 114.9%, *P* < 0.01), and in PVR (reduction of 32.3%, *P* < 0.05).

**Table 3:** Mean changes of parameters at different times of follow-up

Variable	n	Mean (95% CI)	Mean difference from baseline (± SD) (percentage of change)	*P* value
QoL (points)				
1 month	60	1.20 (0.87, 1.53)	− 3.62 (− 3.98, − 3.26) (− 75.1%)	< 0.001
3 months	55	0.80 (0.50, 1.10)	− 4.02 (− 4.38, − 3.66) (− 83.4%)	< 0.001
6 months	53	0.79 (0.51, 1.08)	− 4.00 (− 4.40, − 3.60) (− 83.5%)	< 0.001
12 months	24	0.63 (0.28, 0.97)	− 4.09 (− 4.72, − 3.45) (− 86.6%)	< 0.001
IPSS (points)				
1 month	58	9.21 (7.71, 10.70)	− 12.46 (− 15.39, − 10.52) (− 57.5%)	< 0.001
3 months	53	7.45(6.13, 8.78)	− 13.97 (− 15.64, − 12.30) (− 65.2%)	< 0.001
6 months	54	8.28 (6.77, 9.78)	− 11.91 (− 13.90, − 9.92) (− 59%)	< 0.001
12 months	27	5.89 (4.28, 7.50)	− 12.81 (− 15.57, − 10.06) (− 68.5%)	< 0.001
Qmax (mL/s)				
3 months	50	12.58 (11.44, 13.71)	6.30 (4.66, 7.93) (+ 100.3%)	< 0.001
6 months	49	13.02 (11.96, 14.07)	6.68 (5.41, 7.95) (+ 105.4%)	< 0.001
12 months	27	13.87 (12.10, 15.63)	8.03 (6.07, 9.98) (+ 137.5%)	< 0.001
PV (cm^3^)				
1 month	68	77.96 (67.38, 88.54)	− 15.98 (− 22.86, − 9.09) (− 17%)	< 0.001
3 months	55	71.95 (62.92, 80.97)	− 21.58 (− 30.94, − 12.22) (− 23.1%)	< 0.001
6 months	49	69.90(58.90, 80.90)	− 23.90 (− 34.02, − 13.78) (− 25.5%)	< 0.001
12 months	28	72.89 (59.68, 86.10)	− 34.87 (− 52.68, − 17.06) (− 32.3%)	0.001
PVR (mL)				
3 months	52	96.11 (40.10, 152.12)	− 46.81 (− 134.78, 41.15) (− 32.7%)	0.282
6 months	51	82.65 (43.98, 121.31)	− 65.81 (− 98.05, − 32.52) (− 44.3%)	< 0.001
12 months	26	82.31 (22.29, 142.32)	− 112.56 (− 245.37, 20.26) (− 58%)	0.086
PSA (ng/dL)				
1 month	35	9.02 (1.25, 16.78)	0.92 (− 8.61, 10.46) (+ 11.3%)	0.845
3 months	46	2.54 (1.55, 3.53)	− 4.95 (− 9.51, − 0.40) (− 66.1%)	0.034
6 months	36	2.69 (1.73, 3.64)	− 2.54 (− 3.85, − 1.23) (− 48.6%)	< 0.001
12 months	21	4.30 (1.54, 7.06)	− 6.08 (− 14.93, 2.77) (− 58.6%)	0.166
IIEF (points)				
1 month	48	8.81 (5.72, 11.91)	1.84 (− 0.38, 4.07) (+ 26.4%)	0.102
3 months	49	8.67(5.54, 11.81)	2.84 (0.43, 5.26) (+ 48.7%)	0.022
6 months	44	9.66 (6.10, 13.21)	2.85 (0.14, 5.57) (+ 42%)	0.040
12 months	24	8.88 (4.01, 13.74)	4.13 (0.33, 7.93) (+ 87%)	0.035

*SD* standard deviation, *CI* confidence interval, *IPSS* International Prostate Symptoms Score, *PAE* prostatic artery embolization, *PSA* prostate-specific antigen test, *PVR* post-void residual urine volume, *Qmax* peak urine flow, *QoL* quality of life, *IIEF* International Index of Erectile Function (IIEF), *PV* prostate volume

At 12 months after PAE, PSA decreased 58.6% (6.08 ± 18.91 ng/dL), and IIEF was 4.13 ± 8.80 points (increase of 87%, *P* = 0.0347), but both differences were statistically not significant.

PV measured on MRI showed a statistically significant decrease of 32.3% (*P* < 0.05) from baseline to 12 months post-PAE. Prostatic infarction was present in 64.4% patients at one month after PAE, with a mean volume of 9.4 ± 1.5 cm^3^ (range 0.3–60 cm^3^).

### Clinical Success

Clinical success was obtained in 78.5% of the patients; of whom 45.7% were non-catheterized patients and 54.3% had urinary catheter. Among the non-catheterized patients, there were 6.25% clinical failures in the first month, 0% in the third month, 12.9% in the sixth month, and 8.4% in the twelfth month. Clinical success was higher in patients with bilateral PAE (81% vs 67%; *P* = 0.287).

In patients with urinary retention, removal of the catheter with subsequent spontaneous micturition was achieved in 75% of them (33/44) at first three months after PAE (76% within the first month, and 24% from first to third month). Patients with bilateral PAE achieved higher percentage of catheter removal (76.92 vs 50%; *P* = 0.13). Before PAE, the mean time from urinary catheter insertion was 8.5 months (254.31 ± 375.45 days). After PAE, the mean time to removal of the urinary catheter was 25.8 days (range 1–90 days).

Prior to PAE, 59.46% (22/37) of patients had severe LUTS, and 40.54% (15/37) had moderate LUTS. At 12 months after PAE, 77.78% (21/27) of the patients had mild LUTS, 22.22% (6/27) had moderate LUTS, and there were no patients with severe LUTS (Table 4).

**Table 4:** Time course of LUTS after PAE

	Baseline	1 month	3 months	6 months	12 months
Mild LUTS	0%	44,83%	60,38%	55,56%	77,78%
(IPSS 1–7)	(0/37)	(26/58)	(32/53)	(30/54)	(21/27)
Moderate LUTS	40,54%	46,55%	37,74%	37,04%	22,22%
(IPSS 8–19)	(15/37)	(27/58)	(20/53)	(20/54)	(6/27)
Severe LUTS	59,46%	8,62%	1,89%	7,41%	0%
(IPSS 20–35)	(22/37)	(5/58)	(1/53)	(4/54)	(0/27)

Values between parentheses are number of patients with these symptoms over total number of patients analyzed with IPSS questionnaire

*LUTS* lower urinary tract symptoms, *IPSS* international prostate symptoms score

### Safety

After PAE, 11 patients (13.6%) had 13 minor complications. The distribution of complications according to the modified Clavien–Dindo classification was 30.8% (4/13) grade I, 69.2% (9/13) grade II, with no grade III or IV complications. The complications were urinary tract infection (3/11 patients, 27.3%), urinary retention (3/11 patients, 27.3%), post-embolization syndrome (3/11 patients, 27.3%), erectile dysfunction (1/11 patient, 9%), and femoral artery dissection (1/11 patient, 9%). The patients with urinary retention after PAE were treated by TURP, while patients with urinary tract infection required medical treatment; the remaining adverse events were spontaneously resolved, and all patients recovered without sequelae.

## Discussion

This study has shown statistically significant improvements in subjective (QoL, IPSS, IIEF, pain and satisfaction scores) and objective (Qmax, PVR, PV, prostatic infarct) outcomes in patients treated with PAE using PEGM, achieving a clinical success of 78.5%. These results are comparable to those previously published with PVA [5, 6] and other types of microspheres [4, 19–24], where PAE improved BPH-related LUTS in patients.

Most of the improvements in objective and subjective parameters were observed between the first and third month after PAE, except for PSA. After 3 months, a plateau or slight improvement was observed in all outcomes (Fig. 1). This fact confirms the fast effect of PAE on relief of LUTS described in patients [7, 25]. In addition, PSA increased at one month after PAE, and subsequently decreased up to 66% at 3 months of follow-up compared with baseline data. Early rise in PSA may owe to the initial inflammatory effect associated to prostatic ischemia induced by PAE [26].

The size of PEGM used in the present study was 400 ± 75 µm. Compared to other commonly used microspheres in PAE, such as Embosphere (300–500 µm) and Bead Block (300–500 µm), the tight calibration has a technical advantage of the tighter luminal packing of microsphere once delivered into the target vessels, resulting in greater tissue necrosis [12]. In a report of 186 patients with moderate to severe LUTS who underwent PAE with Bead Block (300–500 µm) [4], the mean IPSS decreased by 12.1 at 1-year follow-up, which is similar to the corresponding data of 12.81 in the present study. However, in the same time period, our data of the mean difference from baseline was superior in QoL score (− 4.09 vs − 1.83), Qmax (8.03 vs 2.33 mL/sec), and PV (− 34.87 vs − 15.2 cm^3^). By contrast, another clinical trial using Embosphere (300–500 µm) in PAE in 88 consecutive patients showed the 1-year outcomes with the mean difference from baseline by − 13.58, -2.9, 9.61 mL/sec, and − 58.11 cm^3^, respectively, in IPSS, QoL score, Qmax, and PV [21]. In addition, Embozene is also a tightly calibrated spherical embolic agent. In a report of preliminary results of PAE in 20 patients [27], clinical success at 6 months was obtained in 76.5%, whereas our data using the same criteria for clinical success showed 87.1% at 6 months after PAE. Nevertheless, to further address and compare the clinical efficacy in PAE among various embolic agents, randomized controlled trials and meta-analyses are needed in the future.

Multiple studies have shown the safe, effective, and durable effect of PAE in patients with urinary retention [28–33]. In those studies, between 70 and 91% of patients with urinary retention were able to void spontaneously after PAE. Carnevale et al. reported a 91% catheter removal rate in 11 men after PAE, with a mean catheter removal time of 12 days [29]. In our study, 54.3% of the patients treated with PAE had urinary retention, with successful removal of the urinary catheter in 75% of them, and a mean time of 25.8 days. This longer time for catheter removal in our study may be explained by the longer period of catheterization prior to PAE (148.5 days).

PAE is a minimally invasive technique showing a lower adverse event profile, less pain, higher satisfaction, and faster recovery time when compared to surgery [20, 34, 35]. No major adverse events were observed in the study, while minor adverse events were reported in 13.6% of patients. The most frequent complications were acute urinary retention, urinary tract infection, and post-embolization syndrome, including dysuria and local pain. A single patient of sexual dysfunction was reported, and there was no incidence of post-procedure retrograde ejaculation or ejaculatory disorders. Our results are comparable with the low rate of complications in the sexual sphere described in other studies [34, 36, 37]. According to published evidence and to our study results, patients with a contraindication to surgery or those who want to preserve ejaculatory and sexual function may benefit from PAE, since it improves urinary symptoms without deteriorating sexual activity due to no urethral manipulation [38].

This observational study had several limitations. Despite being a prospective multicenter study, randomization and comparison with other treatments were not performed. On the other hand, the number of patients was relatively small with a significant lost to follow-up, possibly because of the advanced age of the patients. Furthermore, the IIEF could only be analyzed in a few patients who were sexually active. Another limitation was the medium-term follow-up period limited to 12 months.

In conclusion, PAE with PEGM is a safe and effective technique in the treatment of LUTS secondary to BPH, allowing success in removing the urinary catheter with subsequent spontaneous micturition in a high percentage of patients treated. Comparative studies are needed to assess the potential benefits of this specific embolic agent over the previously reported ones.
