# Correction to: Prostatic Artery Embolization (PAE) Using Polyethylene Glycol Microspheres: Safety and Efficacy in 81 Patients

**DOI:** 10.1007/s00270-023-03371-8

**Published:** 2023-01-24

**Authors:** Iñigo Insausti, Arkaitz Galbete, Vanesa Lucas-Cava, Ana Sáez de Ocáriz, Saioa Solchaga, Raquel Monreal, Antonio Martínez de la Cuesta, Raquel Alfaro, Fei Sun, Manuel Montesino, Fermin Urtasun, José Ignacio Bilbao Jaureguízar

**Affiliations:** 1 Department of Interventional Radiology, Hospital Universitario de Navarra, Pamplona, Spain; 2 Universidad Pública de Navarra–Navarrabiomed–Hospital Universitario de Navarra, Redissec, Instituto de Investigacion Sanitaria de Navarra (IdiSNA), Pamplona, Spain; 3 Endoluminal Therapy and Diagnosis Unit, Jesús Usón Minimally Invasive Surgery Centre, Cáceres, Spain; 4 Department of Interventional Radiology, Clinica Universidad de Navarra, Pamplona, Spain; 5 Servicio Navarro de Salud, Osasunbidea, Pamplona, Spain; 6 Department of Urology. Hospital, Universitario de Navarra, Pamplona, Spain

**Correction to: Cardiovasc Intervent Radiol (2022) 45:1339–1348** https://doi.org/10.1007/s00270-022-03165-4

Please note correction of incorrect “Funding” in the original papers which says that “This study was not supported by any funding”. The “Funding” section should say “Terumo Europe NV has supported this project. The grant funded the study (including open access) and was managed by Navarrabiomed (Pamplona, Spain). The authors were responsible for the design, running and the analysis of the data”.

The original article has been corrected.

